# Novel synthesis of ZnO/PMMA nanocomposites for photocatalytic applications

**DOI:** 10.1038/srep40895

**Published:** 2017-01-18

**Authors:** Alessandro Di Mauro, Maria Cantarella, Giuseppe Nicotra, Giovanna Pellegrino, Antonino Gulino, Maria Violetta Brundo, Vittorio Privitera, Giuliana Impellizzeri

**Affiliations:** 1 CNR-IMM, Via Santa Sofia 64, 95123 Catania, Italy; 2 Department of Physics and Astronomy, University of Catania, Via Santa Sofia 64, 95123 Catania, Italy; 3 CNR-IMM, Z.I. VIII Strada 5, 95121 Catania, Italy; 4 Department of Chemical Sciences, University of Catania, and INSTM UdR of Catania, Viale Andrea Doria 6, 95125 Catania, Italy; 5 Department of Biological, Geological and Environmental Sciences, University of Catania, Via Androne 81, 95124, Catania, Italy

## Abstract

The incorporation of nanostructured photocatalysts in polymers is a strategic way to obtain novel water purification systems. This approach takes the advantages of: (1) the presence of nanostructured photocatalyst; (2) the flexibility of polymer; (3) the immobilization of photocatalyst, that avoids the recovery of the nanoparticles after the water treatment. Here we present ZnO-polymer nanocomposites with high photocatalytic performance and stability. Poly (methyl methacrylate) (PMMA) powders were coated with a thin layer of ZnO (80 nm thick) by atomic layer deposition at low temperature (80 °C). Then the method of sonication and solution casting was performed so to obtain the ZnO/PMMA nanocomposites. A complete morphological, structural, and chemical characterization was made by scanning electron microscopy (SEM), transmission electron microscopy (TEM), energy-dispersive X-ray spectroscopy (EDS), X-ray diffraction (XRD), and X-ray photoelectron spectroscopy (XPS) analyses. The remarkable photocatalytic efficiency of the nanocomposites was demonstrated by the degradation of methylene blue (MB) dye and phenol in aqueous solution under UV light irradiation. The composites also resulted reusable and stable, since they maintained an unmodified photo-activity after several MB discoloration runs. Thus, these results demonstrate that the proposed ZnO/PMMA nanocomposite is a promising candidate for photocatalytic applications and, in particular, for novel water treatment.

Water was taken for granted for centuries, until increasing pollution and reduction of potable water supplies delineated a need to protect our water reserve and develop new technologies to purify water for consumption. For example, it is imperative that pollutants in wastewater effluents, from both industrial and domestic sources, have to be removed before their discharge in the environment. These pollutants are produced as by-products of industrialization and social development, and include conventional and emergent contaminants, such as toxic dye molecules, pharmaceuticals, personal care products, phenols, etc. Conventional water treatment technologies fail in removing the emergent environmental pollutants, generating the need for the development of novel methods.

Nanotechnology offers practical solutions to the problems associated with the production of drinking water and with the protection of natural water for being contaminated^1^,^2^,^3^. At the nanoscale (1–100 nm), materials can display dramatically different physical and chemical properties from their bulk counterparts. Radically, a high surface to volume ratio implies a significant increase in the surface reactivity. Thus, nanotechnology can be used to safeguard our water resources by providing different tools and technology for water treatment and purification.

An innovative purification method involves the combined use of nanostructures and photocatalysts. The heterogeneous photocatalysis is effective in degrading a wide range of refractory organics, eventually mineralizing them in innocuous carbon dioxide and water^4^,^5^,^6^,^7^,^8^,^9^,^10^,^11^. Among the semiconductor photocatalysts, ZnO has received a great interest in the field of photocatalytic technology. This interest has arisen because of its high photocatalytic activity under UV light irradiation (band-gap energy ∼3.3 eV at 300 K), easy growth, low cost, and low environmental impact^12^,^13^,^14^,^15^,^16^,^17^,^18^.

At present, the main technological barrier preventing the commercialization of nanostructured photocatalyst is the post-recovering of the catalyst particles after the water treatment. As a consequence, further efforts consist of the immobilization of the photocatalyst. The incorporation of the active nano-material in a polymeric matrix is a promising approach to overcome this problem. Nanocomposites have attracted considerable research attention in recent years, because they combine low weight and easy formability. Several papers report on nano-ZnO coupled to different polymers, such as: poly(N-isopropylacrylamide)^19^, polyester^20^, hybrid polymers^21^,^22^, polyethylene glycol or Pluronic F127^23^. Among the available polymers, poly(methyl methacrylate) (PMMA) is frequently employed in many applications, since its transparency to visible light, its mechanical properties and chemical stability; it is also a low-cost and hydrophobic polymer suitable for contact with food and drinks. Despite its remarkable properties the PMMA has been rarely coupled with ZnO nanoparticles in order to obtain nanocomposites^24^,^25^. In addition, only few groups tested the photocatalytic performance of ZnO/polymer composites (see as an example ref. 21), and specifically ZnO/PMMA composites have been never tested for applications in wastewater treatment, in spite of the compatibility of PMMA with drinks.

The main objective of this research was to find a simple method to synthesize a novel ZnO/PMMA nanocomposite, usable as raw material in the fabrication of new promising devices for environmental applications. In more detail, PMMA powders were coated with a thin layer of ZnO by atomic layer deposition (ALD), and then ZnO/PMMA nanocomposites were prepared with the method of sonication and solution casting. The composites were deeply characterized by scanning electron microscopy (SEM), transmission electron microscopy (TEM) coupled with energy dispersive X-ray spectroscopy (EDS), X-ray diffraction (XRD), and X-ray photoelectron spectroscopy (XPS) analyses. The photocatalytic performance of the nanocomposites was tested by the degradation of methylene blue (MB) dye, and phenol in water under UV light irradiation. The stability and reusability of the composites was checked for several MB discoloration runs. The effect of the pH of the aqueous solution on the efficiency of dye photo-degradation process was investigated, too.

## Materials and Methods

PMMA (molecular weight: 120000 Da, transition temperature: 105 °C, density: 1.188 g/ml), in a form of powders (0.2–1 mm in diameter) was purchased from Sigma-Aldrich, and used as received. The PMMA powders were covered with a thin layer of ZnO by ALD. The ALD technology is a vapour-based technique like chemical vapour deposition (CVD). In the ALD process the highly reactive precursors are pulsed into the reactor alternatively and separated by purging steps with an inert gas. This results in a stepwise surface-saturated and self-limiting gas-solid reaction mechanism with many advantages, such as good reproducibility, excellent conformality and uniformity over large area, simple and precise control of film thickness, easy composition tuning, and low deposition temperature. These ALD features are very practical in designing and preparing photocatalytic thin films, which enables uniform and conformal deposition also on three-dimensional substrate structures. As a consequence, even porous powders can be easily coated by ALD^26^. The ZnO coating was realized by using the ALD Picosun R-200 Advanced reactor, equipped with a POCA system. The POCA system is a powder coating solution consisting of a quartz glass with a porous separator (pore size from 160 to 250 μm). For this experiment, the POCA system was filled with 2 g of PMMA powders. The deposition of ZnO was performed at 80 °C, lower than the softening/melting point of the PMMA. Diethyl zinc (DEZ, purity 99.9999%) and de-ionized water were used as precursors, while N_2_ was used as carrier and purge gas (purity ≥99.999%). The pulse and purge time were kept constant at 0.1/3/0.1/5 s for DEZ/N_2_/H_2_O/N_2_, using three-times pulses for both DEZ and H_2_O precursor, for a total of 400 cycles. The precursors temperatures were fixed at 22 °C. The synthesized material will be hereafter simply called: “ZnO/PMMA powder”.

In order to quantify the amount of ZnO in the synthesized samples, the PMMA powders were weighed before and after the ZnO deposition, estimating a ZnO percentage in the composite of ∼0.14%.

The ZnO/PMMA nanocomposites were prepared according to the method of sonication and solution casting^27^,^28^. At the beginning, 800 mg of ZnO/PMMA powders were dissolved in 8 ml of acetone. The solution was stirred for 8 hrs, appearing white in colour. Then the mixture was cast into a Petri dish (6 cm in diameter), and dried overnight at 4 °C in a refrigerator so to produce the composite film (about 200 μm in thickness). This film will be hereafter simply called: “ZnO/PMMA composite”. The drying was originally performed at low temperature (4 °C), instead of room temperature^27^,^28^, so to favour the sedimentation of ZnO at the bottom of the Petri dish. After that, the film was peeled off from the Petri dish and used for the morphological, structural and chemical characterization, and the photocatalytic tests. A similar synthesis process was simultaneously performed only with the PMMA powders (i.e., without the ZnO coating), so to obtain a PMMA film used as reference. This film will be hereafter called: “PMMA film”. It is worth to note that both the ALD and the sonication and solution casting processes are easily scalable at industrial level^27^,^29^.

The morphology of the surface of the investigated materials was studied by SEM analyses, with a field emission Zeiss Supra 25 microscope, operating at 3 kV. The samples were previously coated, by sputtering deposition, with a 3 nm thick gold film in order to prevent the electron beam charging from the insulating polymer. In order to perform TEM investigations, the ZnO/PMMA powders were embedded in a liquid epoxy resin. The resin block was then sectioned by ultramicrotomy technique (with a Leica Ultracut UCT), collecting sections of 50–70 nm in thickness on copper mesh “grids” for the TEM observation. The TEM analyses were performed with a JEOL ARM 200 F, working at 200 kV, in conventional bright field (BF) mode. Direct chemical information on the presence of ZnO were acquired by a large area, 100 mm^2^, silicon drift detector EDS spectrometer.

The crystallographic structure was investigated by XRD analyses, with a Bruker D-500 diffractometer (detector scan mode) at 0.8° angle of incidence, and 2θ from 20 to 60°. The XRD spectra were analyzed by the Bruker software suite, including ICSD structure database.

XPS measurements were made with a PHI 5000 Versa Probe II (base pressure of the main chamber 1 × 10^−8^ Pa) using the monochromatized Al Kα radiation and a 5.85 pass energy value. Calibration, procedures to account for the steady-state charging effect, and background removal were already described^30^,^31^.

The photocatalytic activity of the samples was evaluated by the degradation of MB dye in water under light irradiation. Before any measurement, the samples were irradiated by an UV lamp for 60 min in order to remove the hydrocarbons from the sample surface^32^. The samples, in the form of powder (100 mg) or composite (1 cm × 1 cm in size), were thereafter immersed in a 2 ml solution containing MB and de-ionized water (starting concentration of MB: 1.5 × 10^−5^ M). The pH of the solution was fixed at 7.5, using NaOH to control the pH value. The pH value was monitored using a XS instrument pH 8 series pH meter. The dye aqueous solution was left in the dark for 60 or 120 min, so to allow the physical adsorption reached the equilibrium. After these preconditioning steps, the cuvettes, containing the dye aqueous solution and the samples, were covered with a glass so to avoid the evaporation of the solution during the experiment, and were irradiated by an UV lamp (centred at 368 nm, with a full width at half maximum lower than 10 nm, and with an irradiance of ∼2 mW/cm^2^, which simulate the UV irradiance of the Sun on the Earth). The variation of the concentration of the dye in the solution as a function of the irradiation time was measured by the absorbance peak at 664 nm through the Lambert-Beer law^33^, using the spectrophotometer Lambda 45 of the Perkin-Elmer. The irradiation process was carried out for 240 min. The decomposition of the MB dye in the absence of any photocatalyst materials was also checked as a reference. The stability of the nanocomposites was checked by repeating seven times the MB degradation measurements. The effect of the pH of the dye aqueous solution on the photo-degradation of MB was investigated by varying the pH values (from 3 to 12), by adding few drops of HCl or NaOH.

The photocatalytic activity of the ZnO/PMMA composite was also tested for the degradation of phenol, a toxic organic compound, employing the same procedure described above for the MB degradation. The phenol (C_6_H_5_OH), BioXtra (purity ≥99.5%, molecular weight 94.11 g/mol) was purchased from Sigma-Aldrich, and used as received. The starting solution included phenol in de-ionized water, with a concentration of 1.38 mg/l. The pH of the solution was fixed at 7.5. The variation of the concentration of phenol was measured spectrophotometrically (Hach DR 3900 spectrophotometer), using the LCK345 cuvette tests.

## Results and Discussions

The ALD on polymers is a challenging process due to the absence of hydroxyl groups on their surface, that are in principle necessary to activate the growth^26^. As an example, Sinha *et al*.^34^ exploited this surface property investigating TiO_2_ area-selective ALD using PMMA mask as a deposition deactivator. The PMMA inhibiting effect was demonstrated also for ZnO ALD growth^35^. On the other hand, the ALD of Al_2_O_3_ films on polymers (polyethylene, polystyrene, polypropylene, PMMA, and poly(vinyl chloride)) was successfully reported^36^,^37^,^38^. Wilson *et al*.^37^ also proposed a simple model to predict the Al_2_O_3_ deposition without specific chemical species react with tri-methyl aluminium, TMA (i.e., the first precursor). The idea is that the nucleation of Al_2_O_3_ is facilitated by the TMA diffusion into the polymers and the subsequent reaction of the retained TMA with H_2_O (i.e., the second precursor)^37^. The possibility to grow ZnO films on PMMA substrate (both in the forms of bulk and film) by ALD was after reported in literature^39^, also confirming the work by Wilson *et al*.^37^.

In order to perform ALD on thermally-fragile polymers, we deeply investigated low temperature ALD of ZnO^15^. In more details, we deposited ZnO films (with the same thickness) on Si substrates at different deposition temperatures (from 40 to 120 °C). All the ZnO films resulted polycrystalline, irrespective of the low temperature. The film deposited at 80 °C showed the best photocatalytic performance, due to the crystallographic orientation of the film. Once the process was optimized, the ZnO deposition was performed on polyethylene naphthalate (PEN)^15^. The ZnO films showed a significant photocatalytic activity independently of the substrate used, i.e., Si or PEN (for more detailed information, the reader can refer to ref. 15). Based on these results, we reported here the deposition of ZnO layers by low temperature ALD (80 °C) on PMMA powders.

**Figure 1:**
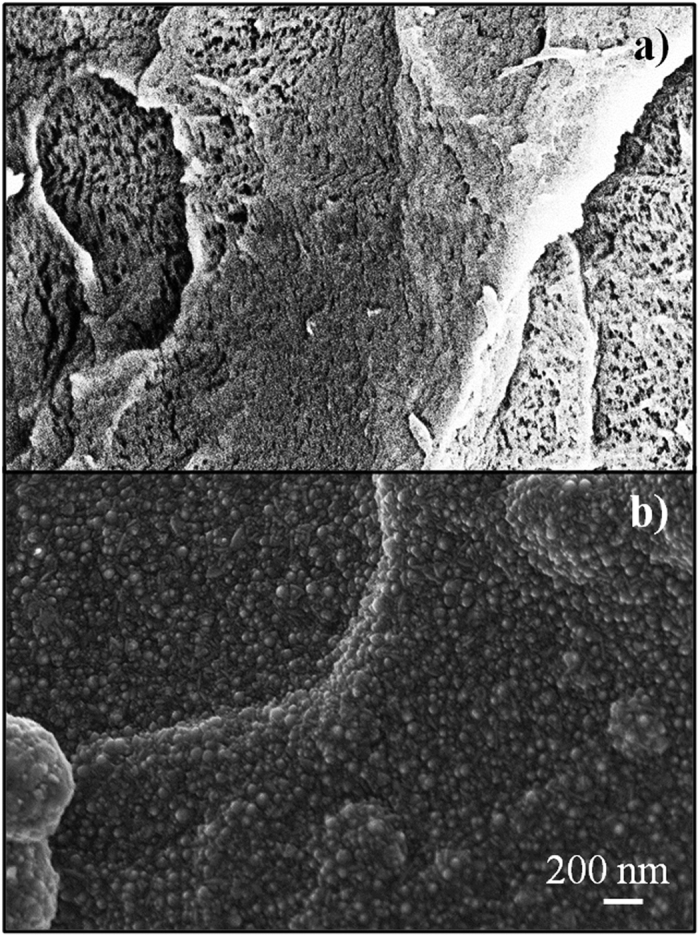
Plan-view SEM images of PMMA powders before (**a**) and after (**b**) the ZnO coating by ALD.

Figure 1(a) and (b) show, respectively, two SEM images in plan-view of the PMMA powders before and after the coating with ZnO by ALD. Figure 1(a) shows the typical texture of PMMA, while Fig. 1(b) shows a granular structure ascribable to the presence of ZnO. Indeed, the literature is unanimous in reporting SEM images showing the surface of ALD ZnO as composed of small grains^40^. In our previous study on low temperature ALD of ZnO film, we showed a ZnO film deposited at 80 °C with a surface full of grains uniformly distributed^15^, similarly to the surface morphology observed in Fig. 1(b).

**Figure 2:**
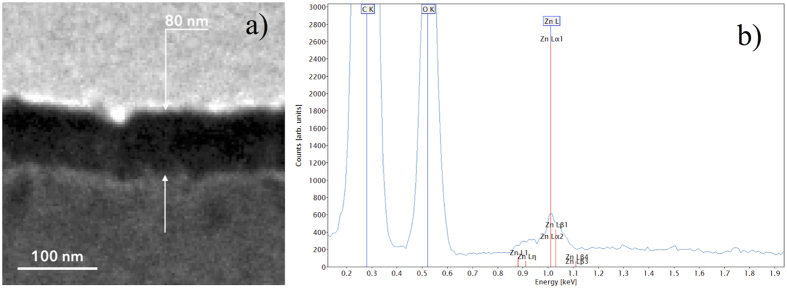
(**a**) Cross-view TEM image of ZnO/PMMA powders. From the bottom to the top, the PMMA substrate and the ZnO film (80 nm thick) can be easily detected. (**b**) EDS spectrum acquired from the same region of the TEM image.

In order to quantify the thickness of the ZnO coating, we analysed by TEM thin (50–70 nm thick) slices of resin containing the ZnO/PMMA powders. Since the PMMA and resin are both insulators, we observed the *ad-hoc* prepared specimen at low magnification conventional BF TEM, and by lowering the beam electron dose as much as possible to minimize the induced electric charge of the specimen itself. Albeit this setup entailed images with a low signal to noise ratio and low resolution, the image resolution showed to be still good for measuring the ZnO thickness. Figure 2(a) reports a TEM image in cross-view of the coated powders. From the bottom to the top, the PMMA substrate and the ZnO film can be easily detected. The ZnO film clearly followed the morphology of the polymer, since the conformality of the deposition. The thickness of the ZnO film resulted ∼80 nm, in good agreement with the thickness of ZnO simultaneously deposited on a Si substrate and measured by spectroscopic ellipsometry. A low-magnification TEM analysis (not shown here) allowed to evaluate the surface of the ZnO/PMMA grains; comparing this value with the one of the ZnO film, we found a ZnO percentage of ∼0.2%, in good agreement with the value obtained by weighing the powders before and after the ZnO deposition (see the Materials and Methods section). The EDS analysis, reported in Fig. 2(b), clearly shows the presence of the L-α and L-β lines, at ∼1 keV, coming from the Zn atoms into the ZnO film, and two K-α lines, at 0.525 keV and 0.277 keV, coming from the O and C atoms, respectively. The oxygen and carbon are present both into the PMMA substrate and into the resin used for the TEM specimen preparation. Being the oxygen not only present into the ZnO film (but also into the PMMA and into the resin) the O X-ray peak intensity is obviously much higher that that one expected for the ZnO film only.

**Figure 3:**
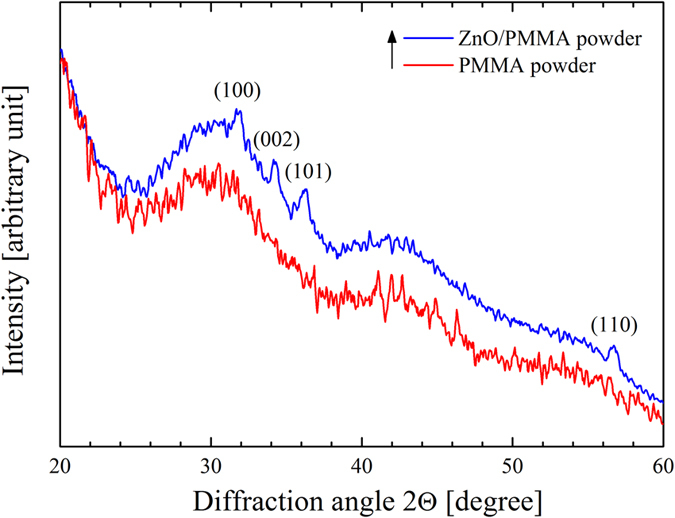
XRD patterns of PMMA powders, and ZnO/PMMA powders.

The crystallographic structure of the powders was investigated by XRD. Figure 3 reports the diffraction patterns for PMMA powders and for ZnO/PMMA powders. The XRD pattern of the PMMA substrate showed broad bands related to the amorphous structure of the polymer. Instead, the ZnO/PMMA powders pattern evidenced, not only the presence of the PMMA matrix, but also the Bragg peaks corresponding to the planes (100), (002), (101), and (110), typical of the wurtzite crystal structure of the ZnO. This means that the ZnO deposited by ALD on the PMMA powders resulted polycrystalline, despite the low deposition temperature (80 °C).

**Figure 4:**
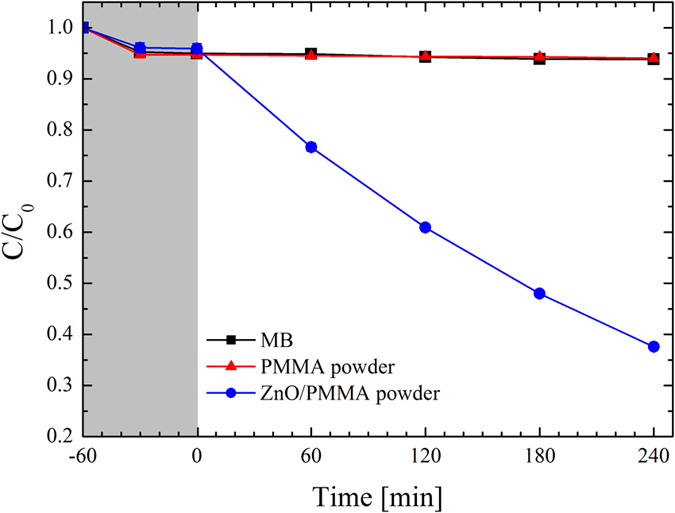
MB photo-degradation under UV light irradiation for three aqueous solutions respectively with: MB (squares), MB with PMMA powders (triangles), and MB with ZnO/PMMA powders (circles).

The photocatalytic activity of the ZnO/PMMA powders was tested by the degradation of the MB dye, a chemical compound commonly used to evaluate the photocatalytic efficiency of the materials. In detail, 100 mg of ZnO/PMMA powders, or merely PMMA powders used as reference, were dispersed into a 2 ml MB and water solution. Figure 4 shows the evolution of the residual concentration of MB versus the time. *C* indicates the concentration of MB at time *t*, while *C*_*0*_ is the starting concentration of MB. The experimental error of the discoloration measurement (1%) is within the symbol size. We tested the MB in the absence of any photocatalyst materials (squares), in the presence of PMMA powders (triangles), and with ZnO/PMMA powders (circles). The first test was performed under dark conditions for 1 hr (reported in grey colour in Fig. 4), in order to evaluate the degree of adsorption of MB on the beaker walls and on the powder surface. Since the MB solution without any sample showed an adsorption of ∼5% (squares in Fig. 4), and there is not any detectable change from this value in the presence of the powders (triangles and circles in Fig. 4), it means that the beaker walls are definitively the responsible for the measured adsorption of MB. Furthermore, it is clear that the investigated materials exhibited a negligible adsorption of MB. When the UV light was switched on, no discoloration was obtained neither with the MB alone (squares in Fig. 4), nor with the PMMA powders (triangles in Fig. 4), as expected. Instead, the synthesized ZnO/PMMA powders induced the degradation of ∼60% of the MB after 4 hrs of light irradiation. The photo-degradation reaction rate (*k*) of the MB contaminant was estimated according to the Langmuir-Hinshelwood model^6^:


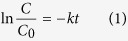


where *t* is the irradiation time. By a simple fitting procedure we found the following value of the photo-degradation reaction rate for the ZnO/PMMA powders: (4.1 ± 0.2) × 10^−3^ min^−1^.

**Figure 5:**
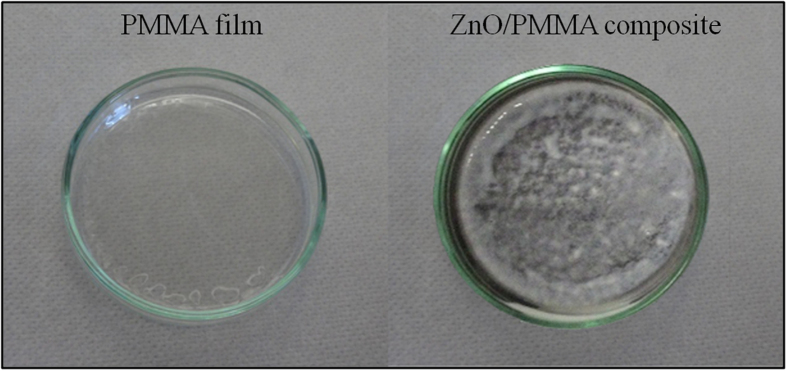
Photographs of PMMA film (left side), and ZnO/PMMA composite (right side) in the Petri dishes.

In the case of aqueous powder dispersion, as reported above, one significant drawback hindering its applications in water treatment is the cost of separating the powder from the water after the treatment. To overcome this obstacle, the immobilization of the photocatalyst powder is a solution. For the mentioned reason, the ZnO/PMMA powders were treated by the method of sonication and solution casting^27^,^28^, so to synthesize the polymeric nanocomposites. Figure 5 reports two photographs of PMMA in the Petri dishes without (on the left) or with ZnO (on the right). The PMMA films were transparent, while the ZnO/PMMA composites appeared white, due to the presence of ZnO.

**Figure 6:**
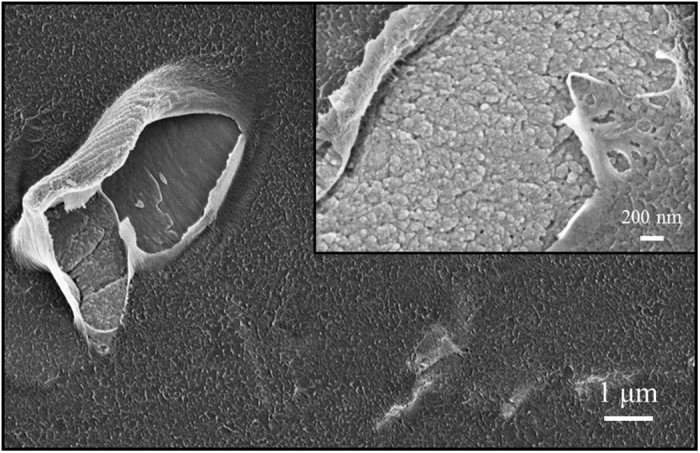
Plan-view SEM analyses of ZnO/PMMA composite. In the inset a higher magnification is reported.

The surface morphology of the ZnO/PMMA composites was investigated by SEM analyses in plan-view, and reported in Fig. 6. The magnification reported in the inset of Fig. 6 showed the typical granularity of ZnO.

**Figure 7:**
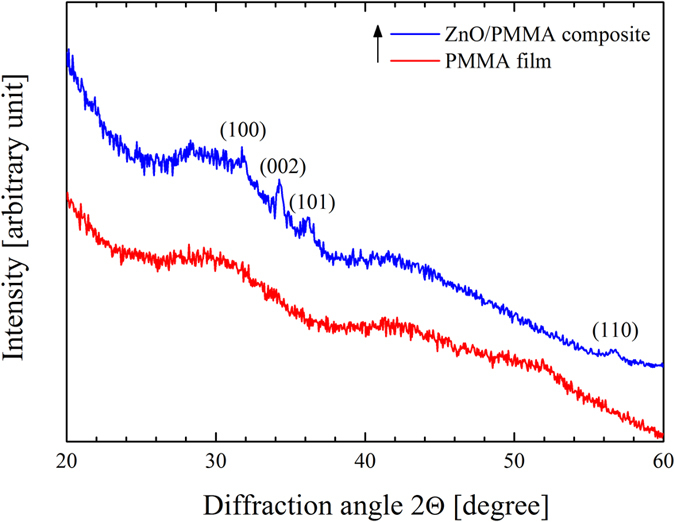
XRD patterns of PMMA film, and ZnO/PMMA composite.

The XRD patterns of the PMMA and ZnO/PMMA composites are reported in Fig. 7. The PMMA film showed broad bands related to the amorphous structure of the polymer, as expected. The ZnO/PMMA composite exhibited, in addition, the wurtzite crystal structure of the ZnO, as observed for the ZnO/PMMA powders (refer to Fig. 3). As a consequence, the sonication and solution casting methodology, applied to synthesize the composite, did not cause any alteration of the crystallographic structure of the ALD ZnO films.

**Figure 8:**
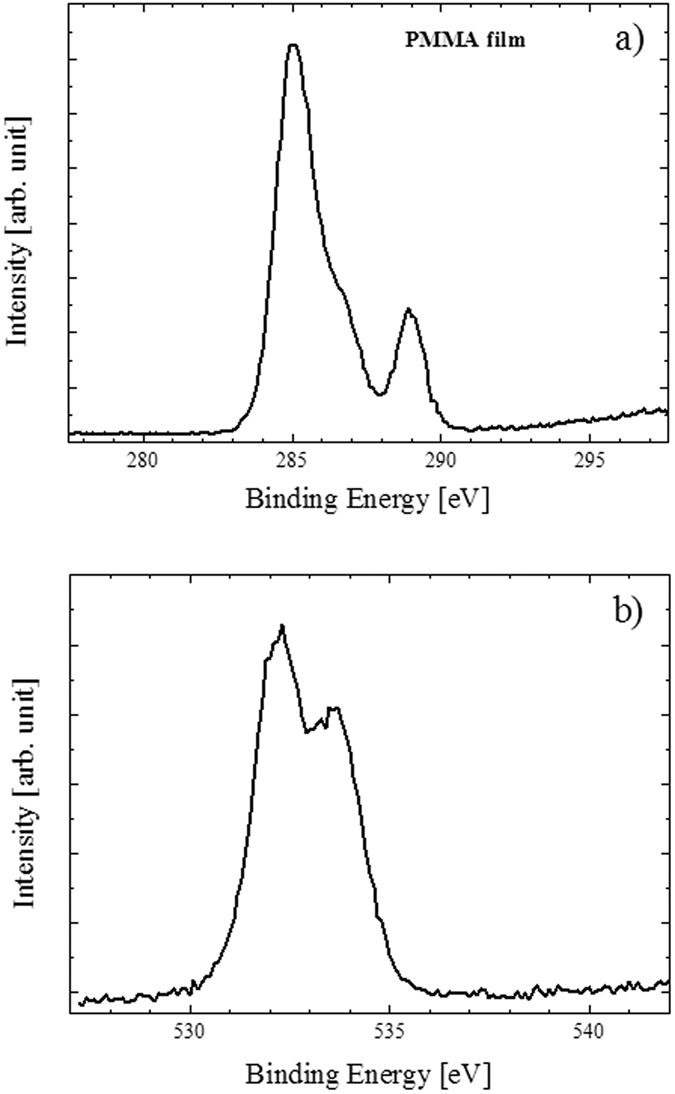
XPS spectra of PMMA film in the C 1 s binding energy region (**a**), and in the O 1 s binding energy region (**b**).

**Figure 9:**
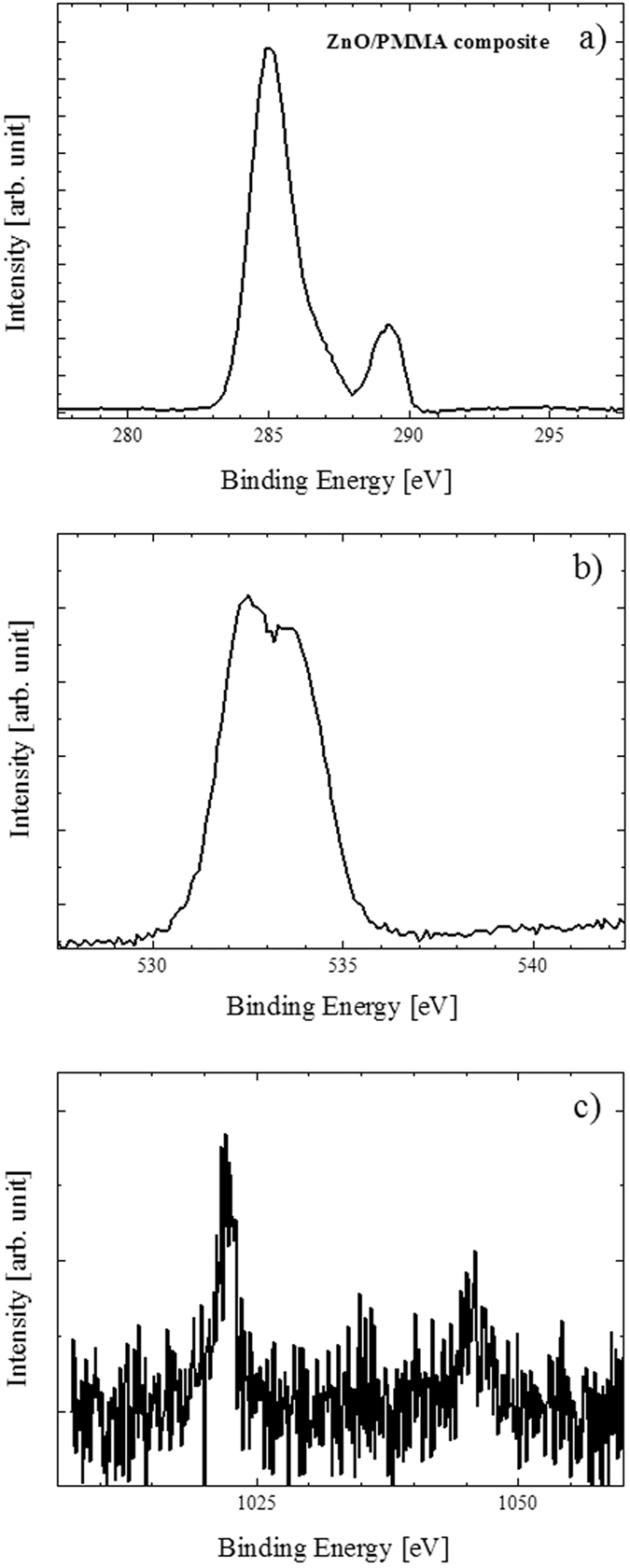
XPS spectra of ZnO/PMMA composite in the C 1 s binding energy region (**a**), in the O 1 s binding energy region (**b**), and in the Zn 2p binding energy region (**c**).

We performed XPS measurements on ZnO/PMMA composites and also on PMMA films, as reference. Figure 8 reports the XPS spectra for PMMA film. The C 1 s spectrum of PMMA (Fig. 8(a)) shows three signals at 285.0, 286.3 and 288.8 eV due to the aliphatic and adventious carbon, to the carbon bonded to one oxygen atom, and to the carbon of the –COO group, respectively. The O 1 s signal of PMMA (Fig. 8(b)) is composed by two peaks at 532.2 and 533.5 eV. These peaks are consistent with the oxygen of the C=O and of the CH_3_-O-C= groups, respectively. These results are in tune with already reported XPS data for PMMA materials^41^,^42^. Figure 9 reports the XPS spectra for ZnO/PMMA composite. The C 1 s spectrum for the ZnO/PMMA composite (Fig. 9(a)) shows again three signals at 285.0, 286.4 and 289.2 eV due to the aliphatic and adventious carbon, to the carbon bonded to one oxygen atom, and to the –COO group, respectively. The most evident difference between the binding energy (BE) values of the present system with respect to the PMMA reference lies in the slight higher BE value of the last ionization peak, that is 0.4 eV at higher BE, thus suggesting some chemical interactions between the –C=O group and the Zn^2+^ ions of the ZnO. The O 1 s spectrum for the ZnO/PMMA composite (Fig. 9(b)) consists of two peaks at 532.5 and 533.5 eV, respectively. Again the higher (0.3 eV) BE of the first oxygen signal confirms the interaction between the –C=O group and the Zn^2+^ ions of the ZnO. Figure 9(c) shows the XPS spectrum for the ZnO/PMMA composite in the Zn 2p energy range. This spectrum is rather noisy in agreement with the very low ZnO content (∼0.2%) with respect to the PMMA. The main 2p_3/2_, 2p_1/2_ spin-orbit components are at 1021.8 and 1045.2 eV, respectively. These features are almost identical to those already reported for related ZnO system^43^,^44^. The chemical formula of the PMMA monomer is (C_5_O_2_H_8_)_n_ and the obtained surface Zn/C concentration ratio is 0.4% which is somewhat higher than the estimated doping level thus suggesting some surface Zn enrichment due to a typical segregation phenomenon. The XPS chemical maps for a representative ZnO/PMMA composite (not shown), acquired on an area of 100 μm × 100 μm, indicated a homogeneous zinc distribution within the material.

**Figure 10:**
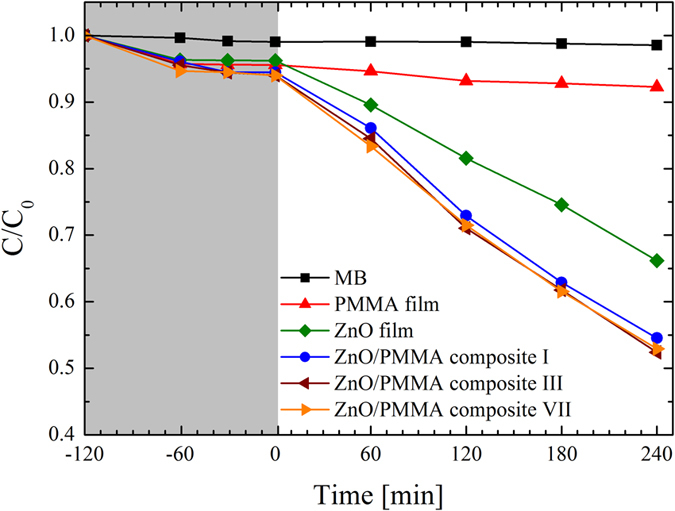
MB photo-degradation under UV light irradiation for four aqueous solutions respectively with: MB (squares), MB with a PMMA film (up triangles), MB with a ZnO film (diamonds), and MB with a ZnO/PMMA composite (circles). The results for the third (left triangles) and seventh (right triangles) test of photo-stability of ZnO/PMMA composites are reported, too.

The photocatalytic degradation of the MB dye caused by the composites is reported in Fig. 10. We tested the ZnO/PMMA composite in comparison with the basic PMMA film (i.e., without the ZnO photocatalyst). In addition, a flat film of ZnO deposited by ALD on a Si substrate was also used as reference. The film thickness was fixed at 20 nm, since we demonstrated that the maximum ZnO photocatalytic activity is reached for ∼20 nm thick film. Indeed, on one hand the photocatalytic performance improves with the film thickness, as a consequence of the higher number of charge carriers generated by the light irradiation in thicker films; on another hand the photocatalysis is strongly affected by the charge diffusion length (for major details the reader can refer to ref. 15). Therefore, we tested four samples: MB aqueous solution without any materials (squares), MB aqueous solution with the PMMA film (up triangles), with the ZnO flat film (diamonds), and with the ZnO/PMMA composite (circles). All the samples were 1 cm × 1 cm in size. The first test was performed in dark conditions and showed a negligible adsorption of the MB on the beaker walls (∼1%, i.e., equal to the experimental error of the discoloration measurements). The investigated materials exhibited a maximum adsorption of ∼5% on their surfaces. This light adsorption saturated after 60 min. No degradation of the MB dye was observed in the absence of ZnO, as expected (see squares for MB, and up triangles for PMMA in Fig. 10), while the presence of the ZnO photocatalyst induced a significant degradation of the dye. In detail, the ZnO flat film degraded ∼30% of MB after 240 min (diamonds), with a photo-degradation reaction rate of (1.7 ± 0.1) × 10^−3^ min^−1^. The best result was obtained thanks to the ZnO/PMMA composite, able to degrade ∼40% of the dye after 240 min (circles), with a photo-degradation reaction rate of (2.6 ± 0.1) × 10^−3^ min^−1^.

It is worth noting that the photo-corrosion of ZnO during the photocatalytic process is a commonly observed phenomenon^45^. As a consequence, the photo-stability of the ZnO in the composite during the photocatalytic process was tested, by repeating the photo-degradation measurements seven times. In detail, at the end of each experiment, the sample was immersed in de-ionized water under UV light irradiation for 2 hrs and then leaved in de-ionized water for 15 hrs, so to remove any possible residues of the dye. The results for the third (left triangles) and seventh (right triangles) test are reported in Fig. 10. This test clearly demonstrated that the ZnO/PMMA composites have an excellent photo-stability over this time range, since they did not show any significant efficiency loss after several experimental runs.

**Figure 11:**
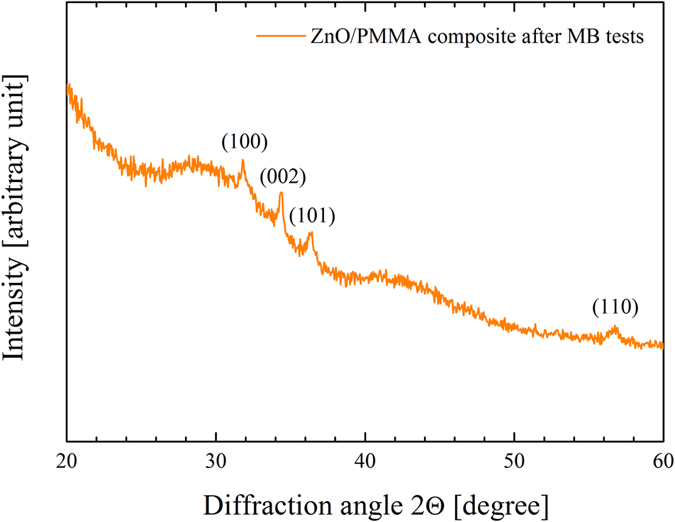
XRD pattern of ZnO/PMMA composite after the seven MB discoloration runs.

The nanocomposites were again characterized by XRD after the photocatalytic tests, in order to verify the stability of the material. Figure 11 reports the diffraction pattern for ZnO/PMMA composite after the seventh MB test. The XRD pattern shows the Bragg peaks corresponding to the planes (100), (002), (101), and (110), typical of the wurtzite crystal structure of the ZnO. Comparing this spectrum with the one obtained before the MB tests (see Fig. 7) we can conclude that the photocatalytic tests did not induced any structural modification of the material.

**Figure 12:**
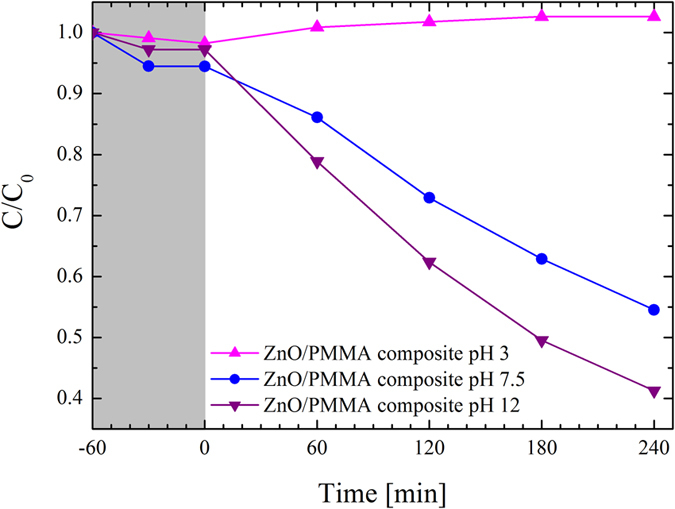
MB photo-degradation under UV light irradiation through ZnO/PMMA composites in MB aqueous solution with different pH: 3 (up triangles), 7.5 (circles), and 12 (down triangles).

The pH of the aqueous solution is one of the most important operating parameter that affect the heterogeneous photocatalysis, since it influences the surface-charge properties of the photocatalyst, and the position of the conductance and valence bands^6^,^7^. Furthermore, the pH of industrial wastewater can be very acidic or basic. As a consequence, the effect of the pH needs to be considered. For these reasons, we carried out an experiment at several pH values of the MB aqueous solution: 3, 7.5, and 12. Figure 12 indicates an increase of the photocatalytic efficiency for higher pH (up triangles for pH 3, circles for pH 7.5, down triangles for pH 12). The decrease in the photocatalytic degradation at acidic pH (null at pH 3) is probably due to the dissolution of ZnO at low pH^46^. On the contrary, at high pH there is an excess of hydroxyl ions, which facilitate the photo-generation of hydroxyl radicals^46^,^47^. In order to verify a possible release of ZnO from the polymer to the solution, after 240 min the composite was removed from the solution at pH 12 and we continued the irradiation with the UV lamp, but no reduction of MB concentration was observed after 60 min. This result demonstrates that ZnO was not released in the MB solution, since we did not detect any photo-degradation.

**Figure 13:**
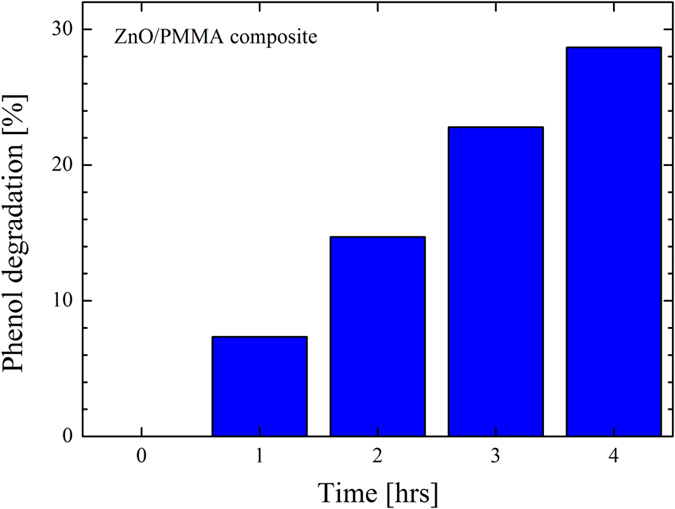
Phenol photo-degradation by ZnO/PMMA composites as a function of the irradiation time.

With the main aim of demonstrating that the ZnO/PMMA composites are also active in the degradation of persistent organic pollutants, the materials were tested by the degradation of phenol in water. The phenols are one of the most important class of pollutants discharged in the environment. The principal sources of phenol pollution in the aquatic environment are wastewaters from paint, pesticides, coal conversion, polymeric resin, petroleum and petrochemical industries^48^. The phenols are considered primary pollutants since they are harmful for organisms also at low concentrations^49^. At high concentration they are even toxic and mutagenic, and may be absorbed through the skin^48^. The performance of the ZnO/PMMA composite in degrading the phenol was monitored with the time, and it is reported in Fig. 13. After 4 hrs under UV light irradiation the photocatalysis induced by the composite removed ∼30% of phenol present in the aqueous solution (at pH 7.5). The photocatalytic response of a control solution only containing phenol and de-ionized water (i.e., without any photocatalyst) was investigated as reference. This blank solution showed a negligible degradation of phenol, as expected.

## Conclusions

In summary, we reported an original, easy, and industrial scalable method to synthesize ZnO/PMMA nanocomposites, by combining ALD and sonication/solution casting process. Commercial PMMA powders were firstly coated with a thin layer of ZnO (80 nm thick) by low temperature ALD, and then the method of sonication and solution casting was applied to produce the ZnO/PMMA composites. The evolution of the materials, from the form of powders to the form of composites was deeply morphologically, structurally, and chemically investigated. In addition, the photocatalytic performance in degrading MB dye and phenol in water was tested under UV light irradiation, showing significant efficiency. The synthesized nanocomposites are also reusable and stable in the water environment, as demonstrated by several MB discoloration tests. The effect of the pH of the dye aqueous solution on the photocatalytic performance of the nanocomposites was investigated, showing an increase of the photocatalytic degradation of MB with increasing pH. Thus, the reported results demonstrate that the incorporation of active ZnO nano-material in a polymeric matrix is a powerful tool to overcome the post-recovery of catalyst particles after the water treatment, opening the route for the commercialization of nanostructured photocatalyst-based technology for an efficient water treatment and purification.

## Additional Information

**How to cite this article**: Di Mauro, A. *et al*. Novel synthesis of ZnO/PMMA nanocomposites for photocatalytic applications. *Sci. Rep.* **7**, 40895; doi: 10.1038/srep40895 (2017).

**Publisher's note:** Springer Nature remains neutral with regard to jurisdictional claims in published maps and institutional affiliations.
